# Hepatitis B virus X protein promotes interleukin-7 receptor expression via NF-κB and Notch1 pathway to facilitate proliferation and migration of hepatitis B virus-related hepatoma cells

**DOI:** 10.1186/s13046-016-0448-2

**Published:** 2016-11-07

**Authors:** Fanyun Kong, Wei Hu, Kai Zhou, Xiao Wei, Yanbo Kou, Hongjuan You, Kuiyang Zheng, Renxian Tang

**Affiliations:** 1 Department of Pathogenic Biology and Immunology, Laboratory of Infection and Immunity, Xuzhou Medical University, Xuzhou, Jiangsu 221004 China; 2 Department of Clinical Laboratory, Suqian People’s Hospital, Nanjing Drum Tower Hospital Group, Suqian, Jiangsu 223800 China

**Keywords:** Hepatocellular carcinoma, Hepatitis B virus X protein, Interleukin-7 receptor, Proliferation, Migration

## Abstract

**Background:**

Interleukin-7 receptor (IL-7R) is involved in the abnormal function of solid tumors, but the role and regulatory mechanisms of IL-7R in HBV-related hepatocellular carcinoma (HCC) are still unclear.

**Methods:**

Gene and protein expression levels of IL-7R were examined in hepatoma cells transfected with hepatitis B virus (HBV) plasmids and in hepatoma cells transfected with the multifunctional nonstructural protein X (HBX). The expression of HBX and IL-7R was measured by immunohistochemical analysis in HBV-related HCC tissues. The role of NF-κB and Notch1 pathways in HBX-mediated expression of IL-7R in hepatoma cells was examined. Activation of IL-7R downstream of intracellular signaling proteins AKT, JNK, STAT5, and the associated molecules CyclinD1 and matrix metalloproteinase-9 (MMP)-9, was assessed in HBX-positive cells with or without treatment with IL-7R short hairpin RNA (shRNA). Additionally, the role of IL-7R in HBX-mediated proliferation and migration of hepatoma cells was investigated.

**Results:**

The expression of IL-7R was increased in hepatoma cells transfected with HBV plasmids; HBX was responsible for the HBV-mediated upregulation of IL-7R. Compared to adjacent tissues, the expression of HBX and IL-7R was increased in HBV-related HCC tissues. Additionally, the relative expression levels of HBX were associated with IL-7R in HBV-related HCC tissues. The activation of NF-κB pathways and expression of Notch1 were increased in hepatoma cells transfected with HBX, and inhibition of NF-κB and Notch1 pathways significantly decreased HBX-mediated expression of IL-7R. The activation of AKT and JNK and the expression of CyclinD1 and MMP-9 were increased in HBX-positive cells. When cells were treated with IL-7R shRNA, the activation of AKT and JNK, as well as the expression of CyclinD1 and MMP-9, were significantly inhibited. Additionally, IL-7R was responsible for HBX-induced proliferation and migration ability of hepatoma cells.

**Conclusions:**

Our data demonstrate that HBX can upregulate IL-7R via NF-κB and Notch1 pathways to facilitate the activation of intracellular pathways and expression of associated molecules, and contribute to proliferation and migration of hepatoma cells.

## Background

Hepatocellular carcinoma (HCC) is a common malignant solid tumor with poor prognosis. According to epidemiological data, most cases of HCC are closely associated with chronic infection with the hepatitis B virus (HBV). In China, over 50 % of HCC patients are HBV carriers [1, 2]. HBV is a double-stranded DNA virus. Its genome comprises the four overlapping open reading frames (ORFs), S, P, C and X, encoding the hepatitis B surface antigen (HBS), preS2, preS1, hepatitis B core (HBC), hepatitis B envelope antigen (HBe), HBV polymerase (HBP), and multifunctional nonstructural protein X (HBX). Despite HBV contributing to the development of HCC, the associated mechanism remains unclear. Increasing evidence indicates that HBX can regulate several cellular processes, including cell proliferation, autophagy, apoptosis, migration, and invasion; HBX also facilitates malignant transformation of liver cells in addition to promoting HCC development [3–5]. Therefore, understanding the mechanisms involved in carcinogenesis mediated by HBX contributes to the development of novel therapeutic approaches targeting HBX and associated factors.

Interleukin-7 receptor (IL-7R) is a heterodimeric receptor containing a specific α chain (IL-7Rα) and a common cytokine receptor γ chain; IL-7R plays a key role in regulating lymphocyte development as well as maintaining survival during differentiation and activation of T cells [6]. Upon engagement with its specific ligand, IL-7, IL-7R activates multiple intracellular signaling pathways, such as Janus kinases (JAK)/signal transducers and activators of transcription (STAT) 5, phosphoinositide 3-kinase (PI3-K), and mitogen-activated protein kinase (MAPK) [7]. Recently, increased expression of IL-7R or/and IL-7 has been observed in hematopoietic malignancies (lymphomas and leukemias) and solid tumors including breast, lung, renal, and colon cancer tissues and cell lines. Additionally, evidence suggests that IL-7R or/and IL-7 play an important role in tumor development and progression [8]. Although previous reports have shown that IL-7R is involved in the development of HCC [9, 10], the exact role of IL-7R in HCC, particularly in HBV-related HCC, is not clear, and the factors responsible for the upregulation of IL-7R in HCC are unknown.

In this study, we examined the expression of IL-7R in HBV-infected hepatoma cells and HBV-related HCC tissues. Further, we investigated the role of HBX in the upregulation of IL-7R, the activation of downstream intracellular pathways of IL-7R, and the expression of associated molecules in inducing cell proliferation and migration. Our data provides novel evidence on the mechanisms involved in HBX-mediated progression of HCC and may aid in the development of therapeutic targets for HBV-associated HCC.

## Methods

### Reagents and cell lines

The HBV plasmid pUC18-HBV1.2 (a vector containing HBV genome) and pcDNA3.1-X (also called pcDNA-HBX, a plasmid containing full-length HBX sequence) were obtained as previously described [11]. The pUC18-HBV1.2-HBX△ plasmid, a vector containing an HBV mutant with a deleted HBX gene, was constructed based on pUC18-HBV1.2 in Transheep Bio (Shanghai, China). The vector containing short hairpin RNA (shRNA) against IL-7R (GGCAGCAAT GTATGAGATTAA), pGPU6/GFP/Neo-IL-7R, and control vectors pGPU6/GFP/Neo with a random interference sequence (UUCUCCGAACGUGUCACGU) were obtained from GenePharma Co, Ltd (Suzhou, Jiangsu, China).

The genes of HBS, preS2, preS1, HBC, HBe, and HBP proteins, encoded by the three overlapping open reading frames (S, C, and P) of the HBV genome, were amplified by polymerase chain reaction (PCR) using the pUC18-HBV1.2 plasmid and cloned into pcDNA3.1 vectors using Nhel and EcoRI or HindIII and EcoRI sites. The primer sequences encoding viral proteins were as follows: HBS (681 bp): CCCAAGCTTATGGAGAACATCGCATCAGGAC and CCGGAATTCTTAAATGT ATACCCAAAGACAAAAGAAAAT. preS1 (1203 bp): CAAGCTTGATGGGAGG TTGGT and CGGAATTCTTAAATGTATACCCAAA. preS2 (846 bp): CCCAAGC TTATGCAGTGGAACTCCACCACTTTCCACCAAAC and CCGGAATTCTTAA ATGTATACCCAAAGACAAAAGAAAAT. HBC (564 bp): CTATAGGGAGAC CCAAGCTGGCTAGCATGGACATTGACCCGTATAAAGAATTTGGAGC and CA CTGTGCTGGATATCTGCAGAATTCCTAACATTGAGATTCCCGAGATTGAGAT CTTC. HBe (651 bp): CTATAGGGAGACCCAAGCTGGCTAGCATGCAACTTTT TCACCTCTGCCTAATCATC and CACTGTGCTGGATATCTGCAGAATTCCTAA CATTGAGATTCCCGAGATTGAGATCTTC. HBP (2544 bp): CTATAGGGAGACC CAAGCTGGCTAGCATGCCCCTATCTTATCAACACTTCCGGAAAC and CACT GTGCTGGATATCTGCAGAATTCTCACGGTGGTCTCCATGCGACGTGCAGAG. The gene sequences encoding viral proteins in the pcDNA3.1 vectors were verified by sequencing.

The anti-HBX mouse polyclonal antibody was purchased from Millipore (Billerica, MA, USA). E-cadherin rabbit anti-human polyclonal antibody, vimentin mouse anti-human polyclonal antibody, and β-catenin mouse anti-human polyclonal antibody were purchased from Ruiying Biological (Jiangsu, China). The p-P65 rabbit anti-human polyclonal antibody, rabbit anti-human P65 polyclonal antibody, p-JNK rabbit anti-human polyclonal antibody, JNK rabbit anti-human polyclonal antibody, p-STAT5 rabbit anti-human polyclonal antibody, STAT5 rabbit anti-human monoclonal antibody, p-AKT rabbit anti-human polyclonal antibody, AKT rabbit anti-human monoclonal antibody, CyclinD1 rabbit anti-human polyclonal antibody, and MMP-9 rabbit anti-human polyclonal antibody were obtained from UCallM Biotech Co, Ltd. (Jiangsu, China). GAPDH mouse anti-human polyclonal antibody, β-actin goat anti-human polyclonal antibody, IL-7R rabbit anti-human polyclonal antibody, and Notch1 goat anti-human polyclonal antibody were purchased from Santa Cruz Biotechnology, Inc. (Santa Cruz, CA, USA). Anti-HBsAg mouse polyclonal antibody was obtained from Bioss Biotechnology (Beijing, China). IgG-HRP goat anti-mouse, IgG-HRP rabbit anti-goat, and IgG-HRP goat anti-rabbit secondary antibodies were purchased from ZSJQ-Bio (Beijing, China). NF-κB pathway inhibitor BAY11-7082 and Notch1 inhibitor GSI-953 were purchased from Sigma-Aldrich (St. Louis, MO, USA). Recombinant human IL-7 was from PeproTech (Rocky Hill, NJ, USA). Trizol reagent and Lipofectamine 2000 were purchased from Invitrogen (Carlsbad, CA, USA). The Cell Counting Kit-8 (CCK-8) was purchased from Dojindo Laboratories (Kumamoto, Japan). Clarity™ Western ECL substrate was purchased from BIO-RAD (Richmond, CA, USA). Transwell cluster plates with 8.0-μm pore were obtained from Corning Costar (Cambridge, Massachusetts, USA). BCA protein kit and crystal violet staining solution were obtained from Beyotime Institute of Biotechnology (Jiangsu, China). TIANScript RT Kit was purchased from TIANGEN Biotech (Beijing, China). LO2, Huh-7, and HepG2 cells were obtained from Cell Bank of Chinese Academy of Sciences. BEL7402 and SMMC7721 were obtained from XiangYa central experiment laboratory, Hubei, China. HepG2.2.15 was a gift from the Peking University Hepatology Institute, Beijing, China.

### Microarray analysis

Huh-7 cells were transfected with the HBV plasmid (Huh-7-HBV) and control plasmid (Huh-7-Mock) and incubated for 48 h. Total cellular RNA was extracted using Trizol reagent following manufacturer’s instructions. The purity of prepared RNA was confirmed using agarose gel electrophoresis. cDNA labeling and microarray hybridization using Affymetrix GeneChip HuGene-1.0ST array platform were performed by the Genminix Informatics Company, Shanghai, China. The microarray data were interpreted, normalized, and log2 scaled using the online analysis tool GCBI (https://www.gcbi.com.cn) and significant differentially expressed genes (DEGs) with fold change ≥ 1.5 were identified. Additionally, the microarray data presented in this study have been deposited in the Gene Expression Omnibus (GEO, http://www.ncbi.nlm.nih.gov/geo/) under the accession number GSE83489.

### Cell transfections

Cells transfections with the pUC18-HBV1.2, pcDNA3.1-X, and pUC18-HBV1.2-HBX△ plasmids using Lipofectamine 2000 were described previously [11]. After Huh-7 and HepG2 cells were transfected with pcDNA3.1-X plasmids for 48 h, 0.4 mg/mL G418 was added into culture medium to select for clones with stable expression of HBX; these clones were then designated as Huh-7-HBX and HepG2-HBX cells.

### Reverse transcription polymerase chain reaction (RT-PCR)

Total cellular RNA was prepared using Trizol reagent per manufacturer’s instructions. Reverse transcription was performed using TIANScript RT Kit. PCR primer sequences for HBX, HBsAg, GAPDH and β-actin, and conditions used for PCR amplification were described previously [11]. Primers for IL-7R and IL-7 were TCTGGAGAAAGTGGCTATGC and CCTGGCGGTAAGCTACATC, ATTGTGATA TTGAAGGTAAAGATG and CATCAAAATTTTATTCCAACA. The conditions for amplification of IL-7R and IL-7 were: 5 min at 94 °C followed by 30 s at 94 °C, 40 s at 50 °C, and 45 s at 72 °C for 38 cycles, followed by a final extension at 72 °C for 5 min. The relative gene expression levels of IL-7R and IL-7 were normalized to the house keeping gene β-actin.

### Western blotting analysis

The protocols for western blot have been described in our previous report [12]. Briefly, proteins extraction was performed using cell lysis and phenylmethanesulfonyl fluoride (PMSF). Protein concentration was measured using the BCA protein kit and adjusted at equal pace. Then, total proteins from different groups were subjected to SDS-PAGE and transferred onto polyvinylidene difluoride (PVDF) membranes. The membranes were blocked using 5 % milk in Tris-buffered saline containing 0.01 % Tween-20 (TBST) for 3 h at room temperature, then incubated with various primary antibodies at 4 °C overnight. Next, the membranes were incubated with HRP-conjugated secondary antibodies for 2 h at room temperature, and protein bands were detected with Clarity™ ECL Western Blot substrate.

### Tumor samples and immunohistochemistry analysis

Forty HBV-related HCC tissues and 10 adjacent tissues were collected at the Department of Pathology, Affiliated Hospital of Xuzhou Medical University, from 2012 to 2015. The study was approved by the ethics committee of Xuzhou Medical University, and informed consent was obtained from all participants. Immunohistochemical analyses were performed using 4-μm formalin-fixed paraffin-embedded (FFPE) tissue sections. First, tissue sections were deparaffinized, rehydrated, and incubated in 0.01 M sodium citrate 5 times for 2 min each time to retrieve the antigens. After incubating with 3 % H_2_O_2_ at room temperature for 15 min, sections were blocked with 10 % goat serum at 37 °C for 30 min, then incubated with specific primary antibodies overnight. Next, the tissue sections were incubated with HRP-conjugated secondary antibodies, followed by detection with DAB (3,3′-diaminobenzidine); double-distilled water was used to terminate the DAB reaction, and then sections were counterstained with hematoxylin.

The detection of relative expression levels of target proteins was based on a semiquantitative system that relied on two parameters: staining intensity and proportion of positive tumor cells as described by Soslow RA et al. [13, 14]. Staining intensity was scored according to the following criteria: 0 (negative staining = no staining), 1 (weak staining = light yellow), 2 (intermediate staining = yellow brown), and 3 (strong staining = brown). The proportion of cells stained was scored as follows: 0 (<10 %), 1 (11–25 %), 2 (26–50 %), 3 (51–75 %), and 4 (>75 %). Total scores were calculated using the following formula: staining intensity score × the score of percentage of positive tumor cells; total scores ranged from 0 to 12. The score of 9–12 was considered strong, 5–8 moderate, 1–4 weak, and 0 was considered negative.

### Cell viability assay

The protocol for cell viability assay has been described previously [12]. Briefly, 100 μL cell suspension of 3 × 10^4^ cells/mL or 2 × 10^4^ cells/mL was placed into 96-well plates with five wells per group. After the plates were incubated for 24, 48, 72, and 96 h, the number of viable cells was tested using the CCK-8. The relative rate of proliferation was determined by optical density (OD) values at 450 nm detected with the ClinBio-128 plate reader (SLT, Austria).

### Plate clone formation assay

The plate clone formation assay has been described previously [12]. Five hundred cells were added to a 6-well plate using three wells per group. After incubation at 37 °C for 2 weeks, the cells were washed twice with phosphate buffered saline (PBS) and stained with the crystal violet staining solution. The clone formation efficiency was calculated as described previously [12].

### Transwell array

The transwell array was performed as described in our previous study [12]. Briefly, 6 × 10^4^ or 4 × 10^4^ cells were resuspended in serum-free medium and placed in the upper part of a transwell plate; the bottom chamber contained 10 % fetal bovine serum (FBS) used as chemoattractant. The cells were then incubated for 24 h. The cells that did not pass through the polycarbonate membrane were scraped off with a cotton swab. The cells that passed through the polycarbonate membrane were designated as cells that had migrated, fixed with 4 % paraformaldehyde, and stained with crystal violet. Finally, the cells were counted as described previously [12].

### Wound healing assay

The cells were plated in 6-well dishes and allowed to reach 90 % confluence in 2 mL of culture medium. Then, a wound was created in the cells using a micropipette tip. Images were taken using a microscope (×400 magnification), and migration distance was calculated as previously described [12].

### Statistical analysis

All data are presented as mean ± SD. Statistical analysis was carried out by Student’s t test, one-way ANOVA, and Mann-Whitney tests where appropriate. A chi-square test was used to analyze the relative expression levels of target proteins in HBV-related HCC tissues and adjacent non-tumor tissues. Additionally, correlations between HBX and IL-7R in HCC tissues were analyzed by Pearson correlation coefficients. A *p* value less than 0.05 was considered significant.

## Results

### HBV induces the expression of IL-7R in hepatoma cells

**Fig. 1:**
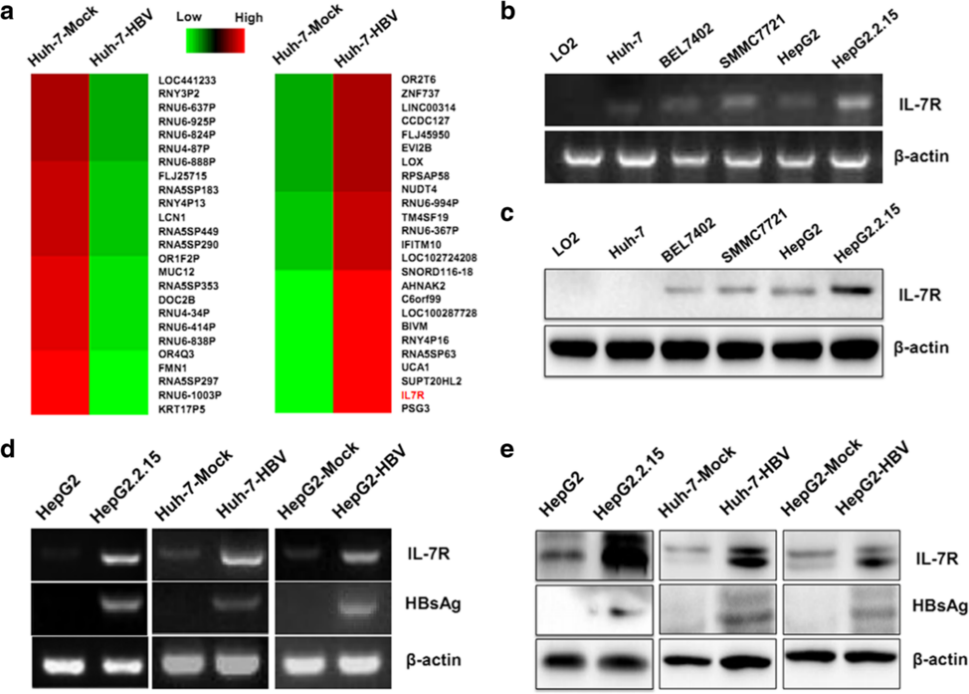
The expression of IL-7R in hepatoma cells transfected with hepatitis B virus (HBV) plasmid. **a** Genetic alteration in Huh-7 cells with fold changes ≥ 1.5 were detected by Affymetrix GeneChip HuGene-1.0 ST array. **b** and **c** The expression levels of IL-7R gene and protein in normal liver cell line and hepatoma cell lines examined by RT-PCR and western blot. **d** and **e** The expression of IL-7R genes and proteins in HepG2 and HepG2.215cells, and in hepatoma cells transfected with the HBV plasmid (Huh-7-HBV and HepG2-HBV) and control plasmid (Huh-7-Mock and HepG2-Mock) for 48 h

To investigate the role of HBV in genetic alteration of hepatoma cells, we first transfected the pUC18-HBV1.2 plasmid and control plasmid into Huh-7 cells. After 48 h, total RNA was extracted and an Affymetrix GeneChip Human Gene 1.0 ST array was used to assess gene expression in both HBV-transfected and control cells. As shown in Fig. 1a, compared to control cells, 25 downregulated genes and 25 upregulated genes with fold change at least 1.5 were observed. Among these genes, the expression of IL-7R was increased in HBV-transfected Huh-7 cells. The expression of IL-7R was also detected in the human normal liver cell line L02 and HCC cell lines including Huh-7, BEL7402, SMMC7721, HepG2, and HepG2.215 cells (HepG2 cells that are stably transfected with a full HBV genome). The expression of IL-7R was not detected in L02 cells, but was found in Huh-7, BEL7402, SMMC7721, HepG2, and HepG2.215 cells (Fig. 1b and c). Among HCC cell lines, HepG2.215 cells expressed the highest levels of IL-7R. We then transfected the pUC18-HBV1.2 plasmid into Huh-7 and HepG2 cells for 48 h to measure the effect of HBV on the expression of IL-7R in HCC cells. The results showed that the expression levels of IL-7R were higher in HBV-transfected HCC cells than in control cells (Fig. 1d and e).

### HBX is responsible for IL-7R expression in HBV-related HCC cells

**Fig. 2:**
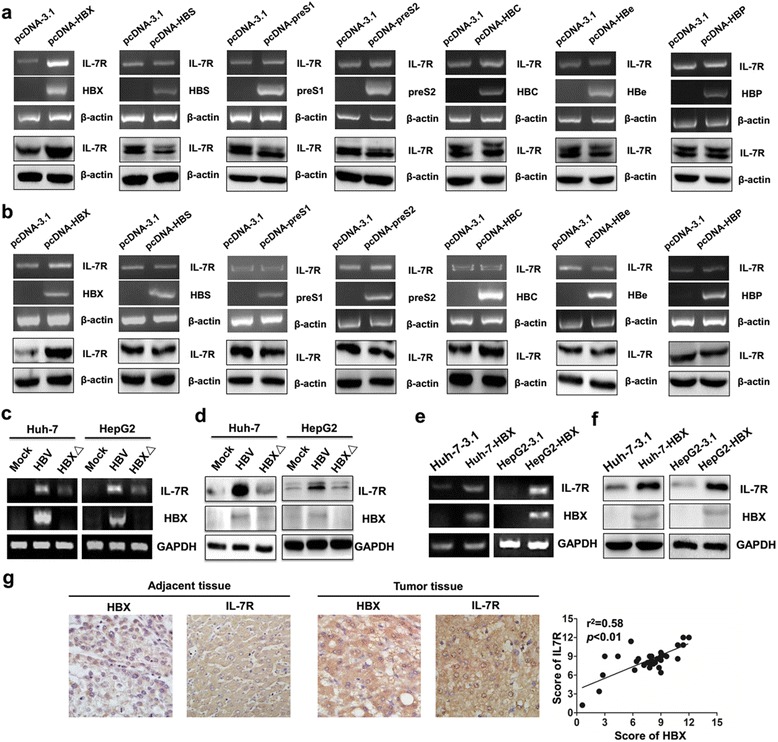
HBX-mediated expression of IL-7R in hepatoma cells. **a** RT-PCR (top) and western blot (down) used to measure the effect of HBX, HBS, preS1, preS2, HBC, HBe, and HBP on the expression of IL-7R in Huh-7 cells. **b** RT-PCR and western blot were used to measure the effect of different viral genes on the expression of IL-7R in HepG2 cells. **c** and **d** The expression of IL-7R gene in hepatoma cells was detected by RT-PCR and western blot after transfection with pUC-18 plasmid (Huh-7-Mock and HepG2-Mock), pUC-18-HBV1.2 plasmid (Huh-7-HBV and HepG2-HBV), and pUC-18-HBV1.2-HBX△ plasmid (Huh-7-HBX△ and HepG2-HBX△) for 48 h. **e** and **f** RT-PCR and western blot were used to examine the expression of IL-7R gene in hepatoma cells stably transfected with pcDNA3.1 plasmid (Huh-7-3.1 and HepG2-3.1) and pcDNA-HBX plasmid (Huh-7-HBX and HepG2-HBX). **g** Immunohistochemistry analysis was used to measure the expression of HBX and IL-7R in HBV-related hepatocellular carcinoma (HCC) tissues (*n* = 40) and adjacent tissues (*n* = 10), as well as correlation between HBX and IL-7R expression in HBV-related HCC tumors

To confirm the HBV proteins responsible for HBV-mediated upregulation of IL-7R, pcDNA 3.1 plasmids containing the genes of seven viral proteins (HBX, HBS, preS1, preS2, HBC, HBe, and HBP) encoded by the four overlapping ORFs (X, S, C, and P) of the HBV genome were transfected into Huh-7 and HepG2 cells for 48 h. The role of different viral genes in the expression of IL-7R was then detected by RT-PCR and western blot. The results showed that only HBX upregulated the expression of IL-7R at the gene and protein levels, while other viral genes had no significant effect on the expression of IL-7R (Fig. 2a and b). HBX is an oncogene that can induce alterations in multiple human genes in HBV-infected hepatoma cells. To further investigate whether HBV-induced upregulation of IL-7R is mainly dependent on HBX, we constructed the HBV mutant plasmid, pUC-18-HBV-HBX△, in which HBX gene is fully deleted based on pUC-18-HBV1.2 plasmids. After transfecting Huh-7 and HepG2 cells with the pUC-18-HBV1.2 and HBV mutant plasmids for 48 h, the expression of IL-7R gene and protein was examined. The results showed that compared with HBV-transfected hepatoma cells, the expression of IL-7R gene and protein were decreased in hepatoma cells transfected with the HBV mutant (Fig. 2c and d). Next, we transfected the pcDNA-HBX vector and control vector into Huh-7 and HepG2 cells and selected the clones (designated as HBX-positive Huh-7-HBX and HepG2-HBX cells) with stable expression of HBX to demonstrate the role of HBX in IL-7R expression. As expected, we found increased expression levels of IL-7R in stable HBX-transfected hepatoma cells when compared with those in control cells (Fig. 2e and f).

To further examine how HBX affects the expression of IL-7R in HCC, we used immunohistochemistry to assess the expression levels of HBX and IL-7R in HBV-related HCC and adjacent tissues. As shown in Fig. 2g, IL-7R was mainly found in the cytoplasm of tumor cells and adjacent tissues. Compared with adjacent tissues, significantly higher expression of IL-7R was found in HBV-related tumor tissues (χ^2^ = 27.2, *p* < 0.01). Additionally, our data indicates that the relative expression level of HBX in HBV-related HCC tissues was significantly higher than that in pericarcinous tissues (χ^2^ = 20.5, *p* < 0.01). Furthermore, positive correlation between the intensity of IL-7R and HBX expression was found in HCC tissues using the Pearson correlation analysis (Fig. 2g). Taken together, these results indicate that HBX induces the upregulation of IL-7R in HCC tissues upon HBV infection.

### HBX up-regulates IL-7R via NF-κB and Notch1 pathways

**Fig. 3:**
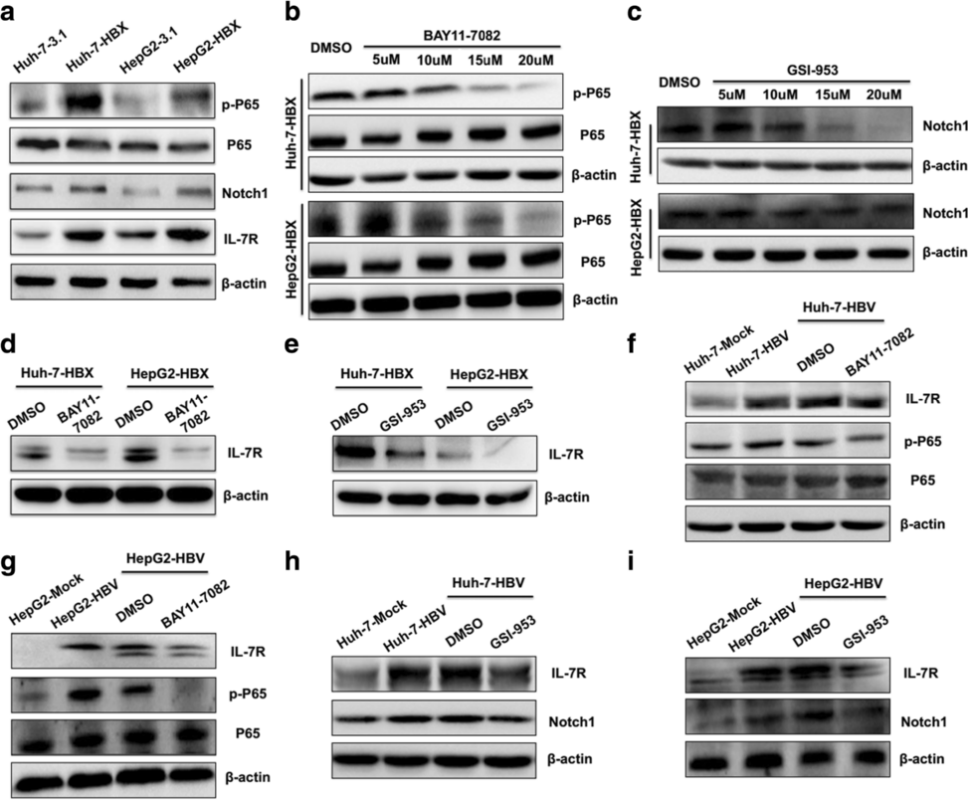
The effect of activated NF-κB and Notch 1 pathways on the expression of IL-7R mediated by HBX. **a** The expression of phosphorylated P65 and Notch1 in hepatoma cells stably transfected with HBX. **b** The selection of a suitable concentration of BAY11-7082 used to inhibit the activation of NF-κB pathway in hepatoma cells stably transfected with HBX. **c** The selection of a suitable concentration of GSI-953 used to inhibit Notch1 expression in hepatoma cells stably transfected with HBX. **d** and **e** The inhibitory effect of 10 μM BAY11-7082 and GSI-953 on the expression of IL-7R in hepatoma cells stably transfected with HBX. **f** and **g** The inhibitory effect of 10 μM BAY11-7082 on IL-7R expression in hepatoma cells transfected with HBV plasmids. **h** and **i** The inhibitory effect of 10 μM GSI-953 on IL-7R expression in hepatoma cells transfected with the HBV plasmid

Next, we explored the potential mechanisms involved in HBX-mediated expression of IL-7R. Miller ML et al. has shown that NF-κB pathway is responsible for the transcription of the IL-7R gene in T cells [15]. Additionally, Wang H et al. indicates that Notch1 pathway is associated with the expression of IL-7R in T cells and T-lymphoblastic leukemia [16]. Based on these reports, we speculated that HBX may activate NF-κB and/or Notch 1 pathway to drive IL-7R expression in hepatoma cells. First, we explored the activation of NF-κB and Notch1 pathways by measuring the phosphorylation levels of the p65 protein, as well as the levels of Notch1, in control and hepatoma cells stably transfected with HBX. The results showed that expression of phosphorylated p65 and Notch1 proteins was higher in HBX-positive cells than that in control cells (Fig. 3a). Next, we measured the half-maximal inhibitory concentration (IC50) of the NF-κB pathway inhibitor BAY11-7082 and Notch 1 pathway inhibitor GSI-953 necessary to suppress NF-κB and Notch 1 pathways in HBX-positive cells. We found that the IC50 for the blockade of both BAY11-7082 and GSI-953 was 10 μM (Fig. 3b and c). Furthermore, when cells were treated with 10 μM BAY11-7082 and GSI-953, the expression of IL-7R was significantly decreased in HBX-positive cells (Fig. 3d and e).

We further tested whether HBV increased the expression of IL-7R via NF-κB and Notch 1 pathways. As shown in Fig. 3f, g, h, and i, HBV activated NF-κB and Notch 1 pathways in both Huh-7 and HepG2 cells. When the cells transfected with HBV plasmids were treated with 10 μ MBAY11-7082 and GSI-953, activation of NF-κB and Notch 1 pathways was inhibited. Additionally, the expression of IL-7R decreased in cells transfected with the HBV plasmid (Fig. 3f, g, h, and i). Together, these results suggest that HBX mediated the increase in IL-7R expression mainly via NF-κB and Notch 1 pathways in HCC cells infected with HBV.

### Increased IL-7R expression mediated by HBX contributes to the activation of intracellular signaling pathways and expression of associated molecules

**Fig. 4:**
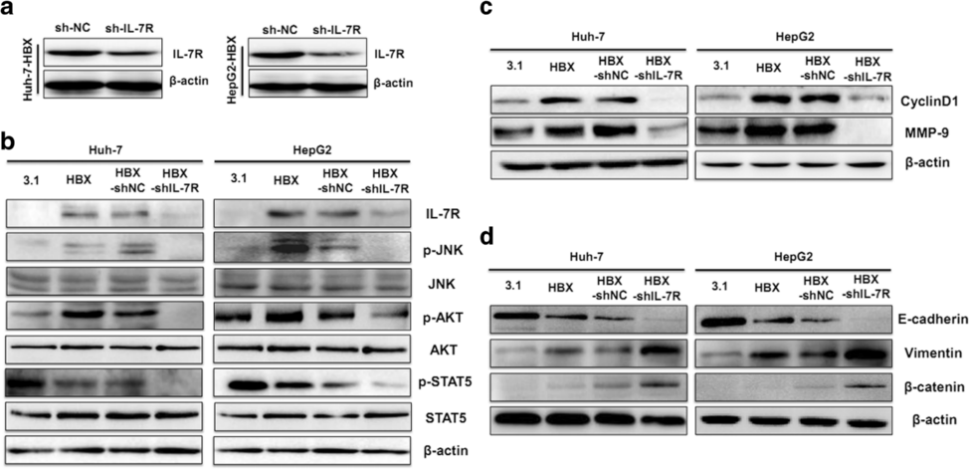
The role of IL-7R in intracellular pathways, molecules, and EMT in HBX-positive hepatoma cells. **a** IL-7R shRNA inhibition of IL-7R expression in hepatoma cells stably transfected with HBX. shNC cells, cells transfected with shRNA control vectors; shIL-7R cells, cells transfected with IL-7R shRNA vectors. **b** The role of IL-7R in the activation of JNK, AKT, and STAT5 in hepatoma cells stably transfected with HBX. HBX-shNC cells, HBX-positive cells transfected with shRNA plasmids. HBX-shIL-7R cells, HBX-positive cells transfected with IL-7R shRNA plasmid. **c** The function of IL-7R on CyclinD1 and MMP-9 in hepatoma cells stably transfected with HBX. **d** The role of IL-7R in the EMT of hepatoma cells steadily transfected with HBX

When IL-7R binds with its ligand, IL-7, it can activate JAK/STAT5, PI3-K, and MAPK pathways [7]; however, whether HBX can activate these pathways through IL-7R is unclear. We constructed IL-7R shRNA vectors to examine the effect of IL-7R shRNA on the expression of IL-7R. As shown in Fig. 4a, IL-7R shRNA significantly inhibited the expression of the IL-7R protein in both Huh-7-HBX and HepG2-HBX cells. Next, the activation of STAT5, AKT (a molecule that belongs to the PI3-K pathway), and JNK (a member of the MAPK pathway) was examined in HBX-positive cells. We found that the phosphorylation levels of AKT and JNK were increased, while the activation of STAT5 declined in HBX-positive cells. Treating HBX-positive cells with IL-7R shRNA inhibited the phosphorylation levels of AKT and JNK (Fig. 4b).

We also examined the molecules downstream of IL-7R, such as CyclinD1, which is associated with cell proliferation [17], and MMP-9, which degrades the extracellular matrix and contributes to cell migration [18], in HBX-positive cells. Our data indicates that the expression of CyclinD1 and MMP-9 was increased in Huh-7-HBX and HepG2-HBX cells. After treating HBX-positive cells with IL-7R shRNA, the expression of CyclinD1 and MMP-9 was significantly decreased (Fig. 4c).

Yang J et al. reported that the IL-7 splicing variant IL-7δ5 contributes to epithelial-mesenchymal transition (EMT) in lung cancer cells [19]. Because the role of IL-7 in EMT is mainly dependent on its interaction with IL-7R, and current studies indicate that HBX uses various mechanisms to induce EMT in hepatoma cells [20], we examined whether IL-7R facilitates EMT in HBX-mediated HCC. As shown in Fig. 4d, HBX could mediate EMT in Huh-7-HBX and HepG2-HBX cells as shown by increased vimentin and β-catenin and decreased E-cadherin. When cells were treated with IL-7R shRNA, the expression of vimentin and β-catenin increased, while E-cadherin decreased significantly, suggesting that IL-7R had a negative role on EMT in hepatoma cells, and that HBX-mediated EMT in hepatoma cells was independent of IL-7R.

### IL-7 stimulates activation of intracellular pathways and increases expression of associated molecules in hepatoma cells via interaction with increased IL-7R mediated by HBX

**Fig. 5:**
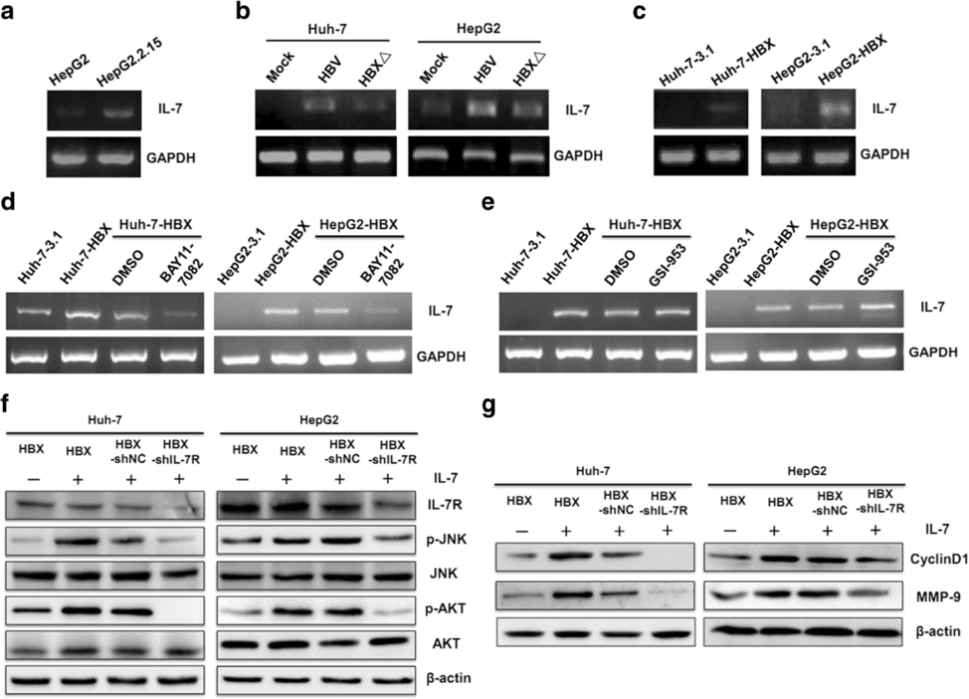
IL-7 gene expression and role of IL-7 in intracellular pathways and molecules in HBX-positive cells. **a** The expression of IL-7 in HepG2 and HepG2.215 cells detected by RT-PCR. **b** The expression of IL-7gene in hepatoma cells detected by RT-PCR after transfection with pUC-18 plasmid, pUC-18-HBV1.2 plasmid, and pUC-18-HBV1.2-HBX△ plasmid for 48 h. **c** The expression of IL-7 genes in hepatoma cells stably transfected with HBX. **d** and **e** The role of NF-κB and Notch 1 pathways in HBX-mediated upregulation of IL-7 in hepatoma cells. **f** The role of IL-7R in the IL-7-mediated activation of intracellular signaling pathways in HBX-positive cells (10 ng/mL). **g** The effect of IL-7R on IL-7-induced expression of associated molecules in HBX-positive cells

Activation of IL-7R is mainly based on its interaction with IL-7. We detected the expression of the IL-7 gene in HBX-positive cells; the results showed that expression of IL-7 gene was increased in HBV-transfected cells compared with that in control cells. Additionally, the increased expression of IL-7 gene mediated by HBV was also mainly relied on HBX (Fig. 5a, b and c). Next, we examined whether the expression of IL-7 mediated by HBX also through NF-κB and Notch 1 pathways. As shown in Fig. 5 d and e, when HBX-positive Huh-7 and HepG2 cells were treated with BAY11-7082, the expression of IL-7 was inhibited. However, when HBX-positive cells were treated with GSI-953, the expression of IL-7 was not significantly changed. Taken together, these results suggest that HBX is capable of inducing the expression of IL-7 through the NF-κB pathway. Additionally, these results imply that autocrine and paracrine IL-7 may activate intracellular signaling pathways and associated molecules in hepatoma cells via interacting with increased IL-7R mediated by HBX. To determine whether IL-7 affects HCC via upregulated IL-7R mediated by HBX, we added the recombinant human IL-7 into the culture medium of HBX-positive cells. The results showed that exogenous IL-7 upregulated the expression of CyclinD1 and MMP-9 and promoted the activation of IL-7R via an increase in intracellular levels of phosphorylated AKT and JNK. When the cells treated with IL-7R shRNA, the increased levels of AKT and JNK phosphorylation, as well as CyclinD1 and MMP-9 expression stimulated by IL-7 were inhibited (Fig. 5f and g). Taken together, these findings suggest that IL-7 could stimulate intracellular pathway activation and increase the expression of associated molecules through IL-7R in HBX-positive cells.

### The proliferation and migration of hepatoma cells mediated by HBX were associated with increased IL-7R

**Fig. 6:**
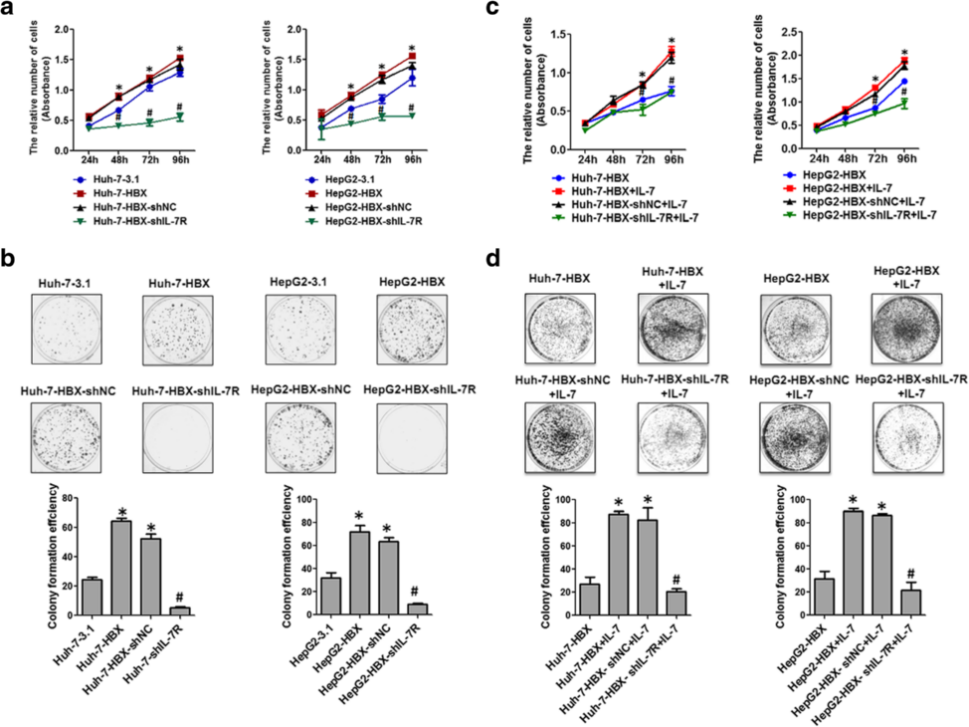
The role of IL-7R in HBX-mediated proliferation of hepatoma cells. **a** The role of IL-7R in HBX-positive proliferation of hepatoma cells as detected by CCK-8. **b** The role of IL-7R in proliferation of HBX-positive hepatoma cells examined by plate clone formation assay. **c** CCK-8 was used to detect the role of IL-7R in the proliferation of HBX-positive hepatoma cells stimulated by IL-7. **d** Plate clone formation assay was used to assess the effect of IL-7R on proliferation of HBX-positive hepatoma cells stimulated by IL-7. *p < 0.05 compared with the cells transfected with control plasmids, ^#^ *p* < 0.05 compared with HBX-positive cells transfected with control vectors

Our previous reports indicate that HBX promotes the proliferation and migration of hepatoma cells [12]. In this study, we found that HBX induces the expression of proliferation-related protein CyclinD1 and migration-associated protein MMP-9. Consequently, we wished to examine whether HBX-mediated IL-7R was involved in the proliferation and migration of hepatoma cells. As shown in Fig. 6a and b, cell viability assay and plate clonal formation assay demonstrated that HBX-positive cells had higher proliferation rates than did the control cells. When cells were treated with IL-7R shRNA, HBX-mediated proliferation in hepatoma cells was decreased. Additionally, upon exposure to exogenous IL-7, proliferation levels of Huh-7-HBX and HepG2-HBX cells were significantly increased compared with those in cells not exposed to exogenous IL-7. During treatment of HBX-positive cells with IL-7R shRNA, the increased proliferation of HBX-positive cells stimulated with IL-7 declined (Fig. 6c and d).

**Fig. 7:**
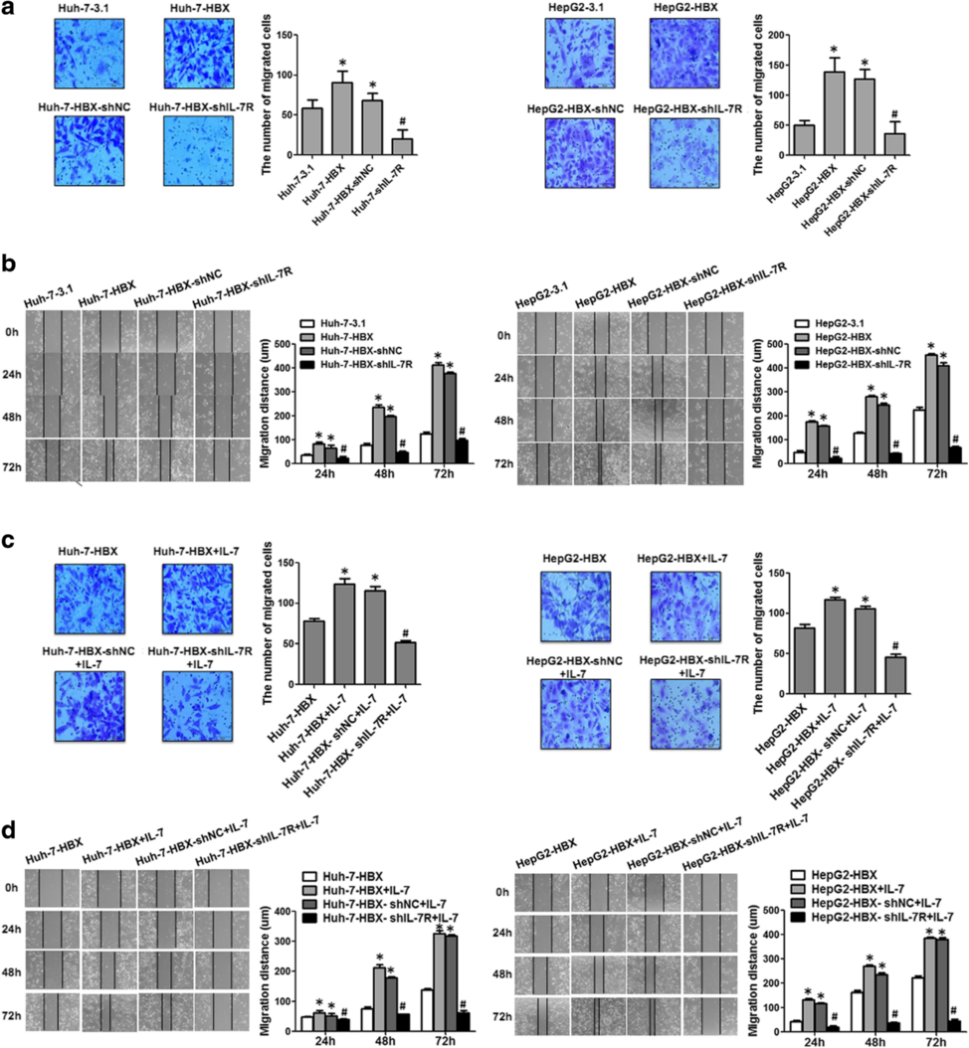
The role of IL-7R in HBX-mediated migration of hepatoma cells. **a** The role of IL-7R in the migration of HBX-positive hepatoma cells as detected by transwell array. **b** The role of IL-7R in the migration of hepatoma cells stably transfected with HBX was examined by wound healing assay. **c** Transwell array was used to detect the effect of IL-7R on migration of HBX-positive hepatoma cells stimulated withIL-7. **d** Wound healing assay was used to detect the effect of IL-7R on the migration of hepatoma cells stably transfected with HBX andstimulated withIL-7. *p < 0.05 compared with the cells transfected with control vectors, ^#^ *p* < 0.05 compared to HBX-positive cells transfected with control plasmids

We next used the transwell array and wound healing assay to explore the function of IL-7R in HBX-mediated cell migration. The results indicated that HBX could enhance the migration of hepatoma cells. When HBX-positive cells were treated with IL-7R shRNA, the migration of hepatoma cells mediated by HBX was inhibited (Fig. 7a and b). In addition, when exogenous IL-7 was added into the culture medium of HBX-positive cells, the migration of these cells significantly increased, while inhibiting IL-7R expression of HBX-positive cells with IL-7R shRNA, the increased migration of HBX-positive cells was inhibited (Fig. 7c and d). Taken together, our results suggest that increased IL-7R is involved in HBX-mediated proliferation and migration of hepatoma cells, and that activation of IL-7R mainly depends on its interaction with IL-7.

## Discussion

Chronic HBV infection is responsible for the development of HCC. The multifunctional regulatory protein HBX plays an important role in hepatocarcinogenesis mediated by HBV infection. HBX can interfere with a variety of cellular processes and uses various molecular mechanisms to impact the progression of HCC [4, 5]. Furthermore, several types of HBX, including HBV whole-X protein (HBwx), which is 56 amino acids longer than HBX, as well as C-terminal truncated HBX, are also implicated in the development of HBV-related HCC [21, 22]. Given the vital role of HBX in HBV-associated hepatocarcinogenesis, defining the mechanisms associated with the development of HBX-mediated HCC will help develop new therapeutic strategies for managing HCC that is complicated by HBV infection. IL-7R is reported to be involved in the functional abnormalities of multiple tumors [8], while the relationship of IL-7R with HBV-related HCC is still unclear. In this study, we found that HBV could increase the expression of IL-7R through HBX in hepatoma cells. Additionally, HBX-mediated increases in the activation of intracellular pathways and expression of associated molecules were related to the upregulation of IL-7R. Furthermore, increased levels of IL-7R contributed to the HBX-induced proliferation and migration of hepatoma cells.

Previous studies have shown that the expression of IL-7R was increased in various tumors such as those of the lung, bladder, and breast [8, 18, 23]. Increased expression of IL-7R was also found to prevent apoptosis and promote proliferation and migration of tumor cells by activating intracellular pathways and upregulating the associated downstream molecules via binding to its ligand, IL-7. Midorikawa Y et al. found that IL-7R gene was downregulated in dedifferentiation of HCC [9]. However, based on high throughput microarrays, Abdel Samee NM et al. suggested that the upregulated IL-7R could be used as a candidate biomarker in HCC [10]. To the best of our knowledge, the role of the regulatory factors associated with IL-7R in HBV-related HCC is still unclear. Using microarray gene expression analysis, we have shown that the expression of IL-7R was upregulated in HBV-transfected hepatoma cells. Additionally, the results of RT-PCR and western blotting analysis further confirmed the results of the microarray, which indicated that HBV was able to enhance the expression of IL-7R in hepatoma cells. Furthermore, we revealed that the HBV-mediated upregulation of IL-7R was mainly dependent on the viral oncogene HBX. Increasing evidence indicates that HBX can induce the alteration of multiple genes to facilitate the development of HCC via various mechanisms [24]; our results suggest that IL-7R may be an essential factor in HBX-mediated proliferation and migration of HCC cells.

Current studies have identified the different signaling pathways responsible for IL-7R expression. Miller ML et al. indicated that NF-κB pathways mediate the transcription of IL-7 receptor, thereby controlling the responsiveness of quiescent naïve T cells to IL-7 [15]. Tian B et al. has shown that the tumor necrosis factor (TNF)-mediated expression of IL-7R is also dependent on NF-κB pathways in HeLa cells [25]. Wang H et al. suggested that Notch1 can bind to distal enhancers of IL-7R and drive its gene expression in T cell development and T-lymphoblastic leukemia [16]. Additionally, RA Sierra et al. have shown that transgenic expression of the Notch 1 intracellular active domain (N1IC) contributes to the increased expression of IL-7R in T cells [26]. Taken together, these results indicate that activation of NF-κB and Notch1 pathways is responsible for IL-7R in cells with different types. Numerous studies have indicated that HBX can stimulate NF-κB and Notch1 pathways to alter the expression of different genes and thereby promote the development of HCC [11, 27–29]. Several studies have reported on the mechanisms responsible for HBX-mediated activation of the NF-κB pathway. It is reported that HBX can enhance the activation of NF-κB pathway via interaction with several intracellular proteins including AIB1 [30], p22-FLIP [31], NEMO [31], TBK1 [32], and VCP [33]. Besides, HBX has been demonstrated to activate NF-κB signaling pathway dependently on other signaling pathways such as PI3-K and Notch 1 [34–36]. With respect to the HBX-induced activation of Notch1, Gao J et al. has shown that HBX upregulated the expression of Notch1 is mainly dependent on p38 MAPK pathway in HCC cells [29]. Additionally, Kongkavitoon P et al. suggested that NF-κB, PI3-K, and MEK1/2 signaling pathways contribute to the activation of Notch1 [28]. Therefore, it is reasonable to speculate that HBX may intensify IL-7R signaling via NF-κB and Notch1 pathways. As expected, we found that the activation of NF-κB pathway and Notch1 expression was increased in HBX-positive cells. Blocking NF-κB pathway and expression of Notch1 with specific inhibitors decreased the expression of IL-7R in HBX-positive and HBV-infected cells.

Activation of IL-7R increased the phosphorylation levels of downstream signaling proteins, including STAT5, AKT, and JNK [7], as well as the expression levels of intracellular molecules, such as CyclinD1 and MMP-9, in tumor cells [17, 18]. Current studies have indicated that HBX is able to activate STAT5 [37], AKT [12], and JNK [11] to regulate various cellular functions. Additionally, HBX can increase the expression of CyclinD1 to promote cellular proliferation [38], and HBX-mediated migration of hepatoma cells is also related to MMP-9 [39]. In our study, we explored whether HBX could activate STAT5, AKT, and JNK, and increase the expression of CyclinD1 and MMP-9 through IL-7R. Our results indicate that HBX could activate AKT and JNK and accelerate the expression of CyclinD1 and MMP-9. Compared with the control cells, the levels of phosphorylated STAT5 in HBX-positive cellular models were lower; these results were not in agreement with previous study from Lee YH et al. [37], who reported that STAT5 could be activated by HBX. This difference may be due to HBX having a different genetic background, which would cause diverse effects in STAT5 activation; further studies are needed to elucidate potential sites in HBX responsible for this discrepancy. Yang J et al. suggested that the IL-7 splicing variant IL-7δ5 is implicated in the EMT of breast cancer cells [19]. Because the role of IL-7δ5 is also dependent on the interaction with IL-7R, it is possible that IL-7R may contribute to the EMT of tumor cells. Although our results have shown that HBX can promote EMT in hepatoma cells, knockdown of IL-7R with IL-7R shRNA did not inhibit, but instead enhanced EMT, in hepatoma cells. These results suggest that IL-7R negatively affected EMT in HCC, and that HBX-mediated EMT was independent ofIL-7R in hepatoma cells.

Based on whole-genome sequencing, Kan et al. reported the amplification of IL-7 gene in 88 HCC tumor tissues, compared to matched adjacent tissues, and even 81 of these tumor tissues were HBV-related HCC [40]. Zhang WY et al. used an oligonucleotide microarray to investigate gene expression profiles in L-O_2_ cells transfected with HBX (L-O2-X cells) and showed that the expression of IL-7 gene was increased in L-O2-X cells [41]. Consistent with Zhang WY et al., our results suggest that HBX can upregulate the expression of IL-7 gene in hepatoma cells. Cattaruzza L et al. suggested that IL-7R can interact with the autocrine and paracrine IL-7 in Hodgkin’s lymphoma cells [42]; therefore, it is reasonable to speculate that the autocrine and paracrine IL-7 in HBX-positive cells can interact with and activate IL-7R. Stimulation with exogenous IL-7 induced activation of AKT and JNK, as well as upregulated the expression of CyclinD1 and MMP-9, in HBX-positive cells. In addition to tumor cells, IL-7 was reported to be expressed in immune cells, fibroblasts, and other stromal cells in vivo [43]. This suggests that in the hepatoma microenvironment, IL-7, secreted by the tumor, as well as by other types of cells, contributes to the abnormal activation of IL-7R in hepatoma cells.

Several studies have demonstrated that IL-7R is involved in proliferation and migration of tumor cells [18, 42]. We investigated whether increased proliferation and migration of hepatoma cells mediated by HBX was dependent on IL-7R. Consistent with our previous reports, HBX could increase the proliferation and migration of hepatoma cells [12]. Additionally, exogenous IL-7 stimulation substantially enhanced the proliferation and migration of hepatoma cells. After treatment with IL-7R shRNA, the proliferation and migration of hepatoma cells, with or without exogenous IL-7 stimulation, were significantly decreased. These data indicate that IL-7R plays an important role in HBX-induced proliferation and migration of hepatoma cells.

## Conclusions

In conclusion, we found that HBV could upregulate the expression of IL-7R via HBX to increase the activation of intracellular pathways and expression of associated molecules, and contribute to the proliferation and migration of hepatoma cells. Our data broadens the current understanding of the role and associated mechanisms of IL-7R in HBV-related HCC and may aid in the exploration of treatment targets for HCC with HBV infection.

## Abbreviations

CCK-8: Cell Counting Kit-8
EMT: Epithelial-mesenchymal transition
HBV: Hepatitis B virus
HBX: Hepatitis B virus X protein
HCC: Hepatocellular carcinoma
IL-7R: Interleukin-7 receptor
MAPK: Mitogen-activated protein kinase
OD: Optical density
ORFs: Overlapping open reading frames
PI3-K: Phosphoinositide 3-kinase
RT-PCR: Reverse transcription polymerase chain reaction
shRNA: Short hairpin RNA
